# Probable tacrolimus toxicity from tibolone co-administration in a woman: a case report

**DOI:** 10.1186/1752-1947-4-276

**Published:** 2010-08-19

**Authors:** Carolyn J Clark, Carmel M Hawley, David W Mudge

**Affiliations:** 1 Department of Nephrology, Level 2, ARTS Building, University of Queensland at Princess Alexandra Hospital, Ipswich Road, Woolloongabba, Queensland, 4102, Australia

## Abstract

**Introduction:**

Tibolone is a synthetic steroid, used with increasing frequency to treat symptoms of menopause, including patients with solid-organ transplants who are taking concurrent immune suppression. To the best of our knowledge, there are no reported drug interactions between tibolone and tacrolimus, one of the principal immune suppressants used in kidney transplantation.

**Case presentation:**

We report the case of a 49-year-old Caucasian woman who had received a kidney transplant and who developed acute kidney injury secondary to tacrolimus toxicity 10 days after starting tibolone therapy. No alternative causes were found. Tibolone is known to be a weak competitive inhibitor of CYP3A4, which is involved in tacrolimus metabolism.

**Conclusions:**

Despite a careful evaluation, no alternative reason was found for the acute kidney injury, and her kidney function returned to the previous baseline within several days of cessation of the medication, and with no other specific treatment. Using the Drug Interaction Probability Scale we conclude that she experienced a probable drug interaction. We believe that transplant clinicians should utilise frequent therapeutic drug monitoring of tacrolimus in patients starting or stopping tibolone therapy.

## Introduction

Tibolone is a synthetic steroid with estrogenic, androgenic and progestagenic properties. It is indicated for relief of the symptoms of menopause in many countries and has been used in Europe for nearly 20 years [1], but is gaining in use elsewhere in the world including Australia. Tibolone use may be increasing following the recent widely-publicised results of the Women's Health Initiative (WHI) study [2], which has resulted in a reduction in the use of conventional estrogen-containing hormone replacement therapy (HRT) [3]. Steroid-induced osteoporosis remains a significant problem in solid-organ transplantation, and it is likely that HRT will continue to be utilized for the prevention of osteoporosis in these patients. Tibolone therapy has been suggested as an alternative to conventional HRT [4] but its role remains unclear, particularly after the recent publication of the Long-Term Intervention on Fractures with Tibolone (LIFT) trial, which showed that although tibolone reduced the risk of fracture and breast cancer, it increased the risk of stroke in older women [5]. We report a case in which a woman who had been the recipient of a kidney transplant with stable allograft function on tacrolimus-based immunosuppression, developed acute kidney injury secondary to tacrolimus toxicity 10 days after starting tibolone therapy. This resolved completely on cessation of the drug. We suggest that this may have been due to a drug interaction between tibolone and tacrolimus, which has not previously been reported.

## Case presentation

A 49-year-old Caucasian woman presented with an acute deterioration in her allograft function seven years after she underwent a deceased-donor kidney transplant for end-stage kidney disease, secondary to autosomal dominant polycystic kidney disease. Her transplant was a three human leukocyte antigen (HLA) mismatch to an unsensitised recipient. The initial therapy was standard immunosuppression at that time (cyclosporin, mycophenolate mofetil and prednisolone). The transplantation was complicated by acute rejection after one year due to steroid withdrawal, which was treated with reinstitution of steroids and conversion to tacrolimus therapy one year later. This was some five years prior to her current presentation. Her serum creatinine concentration then stabilized at 125 μmol/L. She subsequently developed osteoporosis and was commenced on calcitriol and alendronate. She was prescribed HRT in the form of estradiol and norethisterone for both the symptoms of menopause and for protection of her bones. In 2002 after the publication of the WHI study [2], she approached her general practitioner and requested to be withdrawn from HRT, which subsequently occurred over the next two months.

**Figure 1:**
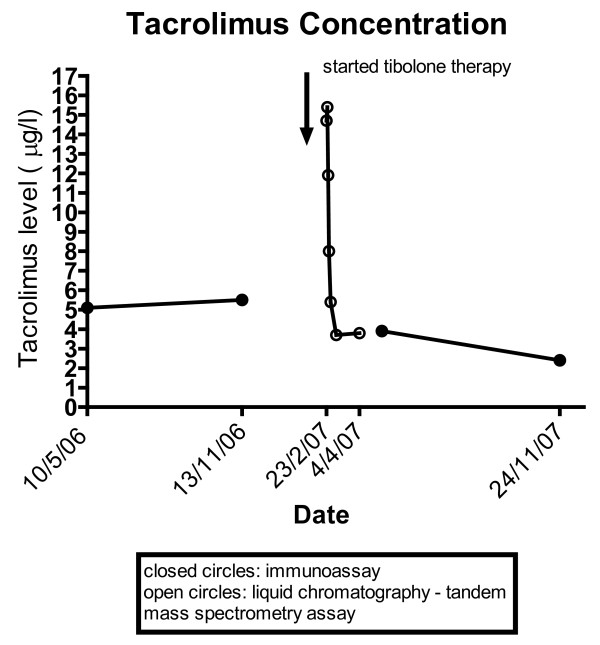
**Whole Blood Tacrolimus Concentration versus Time: note the two different assays used for the tacrolimus concentration reflecting different methodologies used by the external pathology provider and our centre**.

In early 2007, she presented to our transplant centre with lethargy, difficulty sleeping and anxiety over a period of one week. She had no recent illness. Her medications at this time included: tacrolimus 1.5 mg in the morning and 2 mg at night, prednisolone 4 mg daily, mycophenolate mofetil 500 mg twice daily, atorvastatin 20 mg daily, alendronate 70 mg weekly, omeprazole 20 mg daily, citalopram 20 mg daily, calcium carbonate 600 mg daily, calcitriol 0.25 μg daily and folic acid 5 mg daily. The only change to her medications had been the addition of tibolone therapy (2.5 mg per day) for the symptoms of menopause 10 days prior to presentation. On examination she was apyrexial, but was noted to be extremely tremulous and was newly hypertensive with a blood pressure of 144/96 mmHg (previous blood pressure: 118/74 mmHg). She was hyperglycemic with a blood sugar of 12.1 mmol/L, having not been diabetic previously (fasting glucose: 4.5 mmol/L). There was no abnormality found on examination of her cardiovascular, respiratory or gastrointestinal systems. She was found to have worsening graft function with a serum creatinine level of 177 μmol/L (previously 120 μmol/L) and to have tacrolimus toxicity with a level of 17.9 μg/L (liquid chromatography with tandem mass spectrometry (LC-MS/MS) performed at the transplant centre, see Figure 1), having previously been 5.2 μg/L (enzyme immunoassay (MEIA) performed at a local pathology centre). She was serologically positive for BK virus but below the threshold of clinical significance (1 × 10^3 ^at our institution), with a quantitative polymerase chain reaction (PCR) level of 1.7 × 10^2^, which had been noted for several months previously and had not changed.

Management included ceasing tibolone and reducing the tacrolimus dose to 0.5 mg in the morning and 1 mg at night until the concentration reduced to target level, which occurred over the next six days. Her creatinine level slowly returned to baseline over a period of three months, at which time her tacrolimus level was 4 μg/L at a dose of 2.5 mg per day; a similar dosage and drug level to where she had started.

The differential diagnosis of acute kidney injury included tacrolimus toxicity, BK nephropathy, acute rejection and chronic allograft nephropathy. Tacrolimus toxicity was consistent with the acute nature of the presentation, explained all of her symptoms and signs and was borne out by the resolution of her kidney injury as her tacrolimus level and allograft function returned to the previous baseline. It was noted that the baseline and recovery tacrolimus concentrations were measured by MEIA, compared to LC-MS/MS in our centre. However, previous studies have shown that MEIA overestimates tacrolimus concentration in the blood of kidney transplant recipients by 16 to 20 percent [6]. Therefore the different assay methodologies would be likely to underestimate the true size of the effect. BK nephropathy, acute rejection and chronic allograft nephropathy were all considered unlikely due to her clinical presentation and the investigations performed. Although a kidney transplant biopsy was not performed (due to her rapid improvement with reduction in tacrolimus dose and cessation of tibolone), none of these three possibilities would have resolved in this time-frame had they been the true cause of her kidney dysfunction.

The differential diagnosis of tacrolimus toxicity included interaction with new medications, overdose of tacrolimus tablets (either inadvertent or deliberate) and diarrhea. She had no history of a diarrheal illness or loose bowel motions. It was considered unlikely that she accidentally overdosed on the tablets as she was known to be adherent with very consistent blood levels of both tacrolimus and cyclosporin before that. There had been no recent dose changes to cause confusion. The only recent medication had been the tibolone, and although there are no reported drug interactions with tacrolimus and tibolone, we considered it possible that the initiation of tibolone had precipitated the tacrolimus toxicity by some unrecognised mechanism.

## Discussion

Tacrolimus is a calcineurin inhibitor immunosuppressive agent which is metabolized in the liver and the gut by CYP3A4 and CYP3A5 isoenzymes to several metabolites. Tibolone is rapidly metabolized to its three metabolites in the gastrointestinal tract and the liver by 3α/β hydroxysteroid dehydrogenase. Pharmacokinetic studies in three healthy volunteers showed that tibolone was a weak competitive inhibitor of CYP3A4 and CYP2C9, but the authors concluded that tibolone would be unlikely to have a clinically significant effect [7]. Despite that, tibolone has now been shown to interact with warfarin (which is a substrate of CYP2C9 and CYP3A4), requiring dose alterations from 12 to 56 percent in a number of patients [8]. A study of 16 healthy volunteers to investigate this potential drug interaction found an increase in the mean International Normalized Ratio (INR) of 0.4 [9]. However, marked individual variation was noted between subjects, with two of the 16 subjects having a major increase in INR. The authors concluded that it is advisable to monitor for changes in coagulation status during co-administration of tibolone and warfarin.

DIPS has recently been developed to provide a guide to evaluating drug interaction causation in a specific patient [10]. Our case report is considered a 'probable' drug interaction as determined by a DIPS score of 5 or 6 (Table 1). Using this scoring system, our patient experienced a probable drug interaction between tacrolimus and tibolone, and we surmise the likely mechanism is the effect of each drug on her cytochrome P450 system.

**Table 1:** Drug Interaction Probability Scale (DIPS)

DIPS Questions	Answer	Score	Comments
1. Are there previous credible reports of this interaction in humans?	N/A	0	No other case reports exist.

2. Is the observed interaction consistent with the known interactive properties of precipitant drug?	Yes/Unknown	1/0	Given the evidence of tibolone's interaction with warfarin, and the individual variation seen with that interaction, it is reasonable to suggest that in some individuals, there are known interactive properties of the precipitant drug. However, it could also be argued that there is not enough information regarding the mechanism of the interaction between tibolone and warfarin to make this inference.

3. Is the observed interaction consistent with the known interactive properties of the object drug?	Yes	1	Tacrolimus is certainly known to interact with inhibitors of the CYP450 system, of which tibolone is known to be one.

4. Is the event consistent with the known or reasonable time course of the interaction?	Yes	1	Tibolone was started 10 days prior to presentation. As our case report had been stable for some time on tacrolimus, there was no baseline blood test. However, the development of the symptoms consistent with acute tacrolimus toxicity occurred within a reasonable time course of the start of tibolone use.

5. Did the interaction remit upon dechallenge of the precipitant drug with no change in the object drug?	N/A	0	Tibolone was ceased immediately, but due to the effects of the tacrolimus toxicity (acute kidney injury in a transplant patient) the dose of tacrolimus was also altered.

6. Did the interaction reappear when the precipitant drug was readministered in the presence of continued use of the object drug?	N/A	0	She was reluctant to re-challenge with tibolone.

7. Are there reasonable alternative causes for the event?	No	1	As discussed above, alternative causes were looked for and none were found.

8. Was the object drug detected in the blood or other fluids in concentrations consistent with the proposed interaction?	Yes	1	See Figure 1 for tacrolimus concentrations.

9. Was the drug interaction confirmed by any objective evidence consistent with the effects on the object drug (other than drug concentrations)?	Yes	1	She was hyperglycemic and hypertensive, both known effects of tacrolimus toxicity. She also displayed a tremor, and had symptoms of anxiety.

10. Was the interaction greater when the precipitant drug dose was increased or less when the precipitant drug dose was decreased?	N/A	0	The tibolone was ceased at presentation and alternate doses were not used.

## Conclusions

Given the polymorphisms in the cytochrome P450 metabolic pathway and the potential for a wide variation in the metabolism of these two drugs, there is a strong likelihood that other patients may experience a similar interaction. However, given pharmacogenetic variability, not all patients would be expected to be affected. Primary care physicians may not seek the advice of transplant specialists for treatment not directly related to the transplant, and given that there are no reports in the literature, would not expect a complication such as this. We believe that all clinicians (transplant and primary care) should be aware of this probable drug interaction and should ensure monitoring of tacrolimus concentration within three days of commencement of tibolone. Prophylactic dose reduction of tacrolimus is not advised, as it is not likely that all patients will be affected.

## Consent

Written informed consent was obtained from the patient for publication of this case report and any accompanying images. A copy of the written consent is available for review by the Editor-in-Chief of this journal.

## Competing interests

The authors declare that they have no competing interests.

## Authors' contributions

CC wrote the manuscript. CH reviewed our case report and provided her data. DM edited the manuscript and provided overall direction. All authors read and approved the final manuscript.
